# Shape‐Anisotropic Assembly of Protein Nanocages with Identical Building Blocks by Designed Intermolecular π–π Interactions

**DOI:** 10.1002/advs.202305398

**Published:** 2023-10-23

**Authors:** Xuemin Chen, Tuo Zhang, Hanxiong Liu, Jiachen Zang, Chenyan Lv, Ming Du, Guanghua Zhao

**Affiliations:** ^1^ College of Food Science & Nutritional Engineering Beijing Key Laboratory of Functional Food from Plant Resources China Agricultural University Beijing 100083 China; ^2^ School of Food Science and Technology National Engineering Research Center of Seafood Dalian Polytechnic University Dalian 116034 China

**Keywords:** crystal structure, π–π interactions, protein array, protein design, shape tunability

## Abstract

Protein lattices that shift the structure and shape anisotropy in response to environmental cues are closely coupled to potential functionality. However, to design and construct shape‐anisotropic protein arrays from the same building blocks in response to different external stimuli remains challenging. Here, by a combination of the multiple, symmetric interaction sites on the outer surface of protein nanocages and the tunable features of phenylalanine‐phenylalanine interactions, a protein engineering approach is reported to construct a variety of superstructures with shape anisotropy, including 3D cubic, 2D hexagonal layered, and 1D rod‐like crystalline protein nanocage arrays by using one single protein building block. Notably, the assembly of these crystalline protein arrays is reversible, which can be tuned by external stimuli (pH and ionic strength). The anisotropic morphologies of the fabricated macroscopic crystals can be correlated with the Å‐to‐nm scale protein arrangement details by crystallographic elucidation. These results enhance the understanding of the freedom offered by an object's symmetry and inter‐object π−π stacking interactions for protein building blocks to assemble into direction‐ and shape‐anisotropic biomaterials.

## Graphical abstract

A strategy is proposed to design diverse shape‐anisotropic protein arrays in response to different stimuli by utilizing the tunable nature of π–π stacking interactions and the symmetric distribution of multiple interaction sites on protein nanocage surfaces. These findings provide valuable insights into the assembly of direction‐ and shape‐anisotropic biomaterials using protein building blocks.

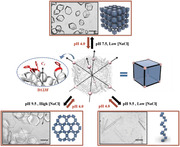

## 1 Introduction

In Nature, shape tunability is imperative to perform a plethora of complex functional activities in response to external stimuli, for example, blooming of flowers, flight of birds, growth of pseudopod in Amoeba proteus.^[^ ^1^ ^]^ The engineering of self‐shaping “smart” architectures or devices that can morph in a controlled manner as living systems has generated considerable interests in the field of nanoscience and nanotechnology^[^ ^2^ ^]^ because of immense scientific and economic value. At a molecular level, a single viral capsid protein fold can be evolved to form multiple oligomeric states with different symmetries,^[^ ^3^ ^]^ and similarly, clathrin has the ability to self‐assemble into several regular assemblies, such as 78‐mer, 108‐mer, and 180‐mer.^[^ ^4^ ^]^ However, to construct protein architectures whose structure and shape‐shifting could be modulated in a controlled manner remains challenging.

During evolution, proteins as the most versatile building blocks have acquired self‐assembly properties to generate a variety of large, complex, and symmetric 0D, 1D, 2D, and 3D architectures,^[^ ^5^ ^]^ endowing their hosts with plenty of functions. Among all naturally occurring proteins, ubiquitous protein nanocages have drawn considerable attention due to their unique functions, such as viral capsids in nucleic acid storage and transport,^[^ ^6^ ^]^ clathrin cages in endocytosis,^[^ ^7^ ^]^ carboxysomes in CO_2_ fixation,^[^ ^8^ ^]^ Dps in DNA protection,^[^ ^9^ ^]^ and ferritin in iron metabolism.^[^ ^10^ ^]^ More importantly, these protein nanocages possess promising physicochemical and biochemical properties including high symmetry, solubility and stability, monodispersity, and ease of genetic and chemical manipulation, so they have been explored as biotemplates for the preparation of inorganic and organic nanomaterials, and the encapsulation of guest molecules with various potential applications.^[^ ^11^ ^]^ To build biomimetic protein nanocages or their assembled superlattices that rival the size and functionality of natural protein nanocage assemblies, different strategies such as de novo design,^[^ ^12^ ^]^ fusion protein,^[^ ^13^ ^]^ directed evolution,^[^ ^14^ ^]^ and key interface redesign^[^ ^15^ ^]^ have been built to create a variety of artificial protein nanocages or 2D and 3D crystalline protein nanocage arrays; some of these engineered protein assemblies are competitive owing to their excellent mechanical and functional properties. However, unlike atoms with precise bond geometries and binding energies, control over mesoscale protein crystal growth direction and morphology are grant challenges as the vast and heterogeneous protein–protein interactions make the crystal contacts and lattice packing arrangements more unmanageable, which allows little external control over protein assembly with bulk‐scale protein architectures. Most of the designed crystalline protein nanocage materials are devoid of direction‐controlled assembly and dynamic conversion, which is a key characteristic of many molecular and macroscopic materials/device.

It has been well‐established that noncovalent protein–protein interactions (PPIs) are mainly involved in the formation of complex protein architectures. Such noncovalent interactions at intermolecular interfaces are exquisitely, tightly controlled, which define the orientation, dimension, and geometry of protein assemblies.^[^ ^5^, ^11^ ^]^ Among reported strategies, multiple interactions such as metal coordination bonds,^[^ ^15a,b^ ^]^ disulfide bonds,^[^ ^16^ ^]^ hydrogen bonds,^[^ ^17^ ^]^ electrostatic attractions,^[^ ^18^ ^]^ and hydrophobic interactions^[^ ^19^ ^]^ have been developed, but aromatic amino acid residues involved π−π stacking interactions have been largely unexplored to dedicate the assembly of protein blocks into highly ordered protein architectures, especially those with self‐shaping properties. Similar to other noncovalent interactions, π–π stacking interactions coming from naturally occurring aromatic amino acid residues such as phenylalanine (Phe), tyrosine (Tyr), tryptophan (Trp), and histidine (His) play important roles in folding or self‐assembly of protein architectures.^[^ ^20^ ^]^ Unlike other noncovalent interactions, the geometry of π−π interactions between two aromatic residues, especially Phe‐Phe involved π–π interactions, are diverse and tunable, which can be broadly classified into at least three categories: edge‐to‐face (T‐shape), parallel offset, and parallel face‐centered stacked^[^ ^21^ ^]^ as shown in Figure S1 (Supporting Information). It has been known that π−π stacking interactions are mainly driven by electrostatic interaction and hydrophobic effect (the desolvation effects),^[^ ^21^, ^22^ ^]^ and thus their relative energy ordering and geometry are highly pH‐dependent^[^ ^23^ ^]^ and are also affected by ionic strength in the solution.^[^ ^24^ ^]^ On the other hand, the magnitude and charge of protein building blocks can also be adjusted by pH and salt concentration. Even subtle differences in these factors can result in distinct assemblies.^[^ ^25^ ^]^ Thus, the interesting characteristics of the π–π stacking interactions and protein building blocks could provide a solution to design intermolecular interactions to trigger protein assemblies in a condition‐responsive manner. However, so far, the aforementioned high flexibility of aromatic resides involved π–π interactions in anisotropic self‐assembly of protein building blocks in response to external stimuli has been largely unexplored.

**Figure 1:**
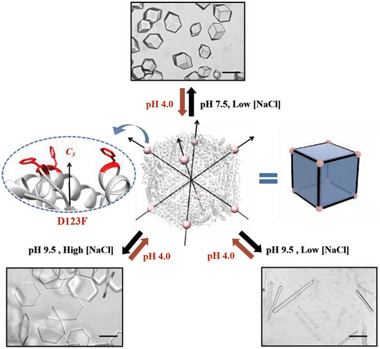
Schematic illustration of shape tunability between different crystalline protein nanocage arrays through designed Phe‐Phe interactions in response to external stimuli. The side chain of Phe resides protrudes on the outside surface of the protein cage, which facilitates intermolecular interactions. The scale bar of optical micrographs is 100 µm.

Here, by taking advantage of the tunable features of π−π stacking interactions and the symmetric distribution of multiple interaction sites on the exterior surface of protein nanocages, we introduce an engineering strategy that could be used for fabrication of crystalline protein nanocage arrays whose assembled orientation can be regulated by adjusting solution conditions. The first step of this strategy is to analyze the symmetry of a targeted protein nanocage building block with crystal structural information as a guide to determine its symmetry axes such as *C_3_*, *C_4_*, and *C_5_*. The second is to identify one polar amino acid residue lining along one of these symmetry axes nearby the protein outer surface, followed by mutating this polar residue to Phe to prepare Phe‐decorated protein nanocages. The last step corresponds to finding appropriate solution conditions (pH and ionic strength), which facilitates self‐assembly of the above Phe‐decorated protein nanocages into shape‐ansiotropic crystalline protein arrays. In order to achieve the proof‐of‐concept objectives, we chose a 24‐mer human H‐chain ferritin (HuHF) nanocage as starting materials; subsequently its original Asp residues nearby the *C_3_* symmetry axes were mutated into Phe, and the resulting mutant is referred to as 3FF. We applied this strategy to create 1D, 2D, and 3D crystalline protein nanocage arrays by using the same 3FF building blocks under different solution conditions, while realizing shape tunability of these created protein arrays through discrete nanocages as intermediates (**Figure** **1**).

## 2 Results and Discussion

### 2.1 Design and Characterization of 3D Crystalline Protein Nanocage Arrays

Among the protein nanocages, ferritin nanocage, especially recombinant human H chain ferritin (rHuHF), has one major advantage: high selectivity for cancer cells which overexpress the H‐type ferritin receptor, TfR1,^[^ ^26^ ^]^ so it has emerged as a class of drug delivery vehicles and imaging agents.^[^ ^5d^ ^]^ Additionally, rHuHF is a 24‐mer homopolymer with high symmetry, stability, and water solubility, which can be easily overexpressed and purified from Escherichia coli. Based on these promising properties, we chose rHuHF as building blocks for the construction of highly ordered protein assemblies.

Ferritin shares the highly conserved architecture that usually consists of 24 four‐α‐helix bundle subunits assembling into a quasi‐spherical shell with octahedral (432) symmetry, so it can be considered as a cubic‐like protein as shown in Figure 1. One ferritin molecule consists of six *C_2_* rotation axes, four *C_3_* rotation axes, and three *C_4_* rotation axes. From a standpoint of assembly, each ferritin molecule consists of three types of interfaces responsible for its cubic‐like structure, namely, *C_2_*, *C_3_*, and *C_4_* (Figure S2, Supporting Information). To create dynamic, transmutable protein nanocage arrays in this study, we selected the outer surface around the *C_3_* axes of ferritin to introduce intermolecular π−π stacking interactions, because the *C_3_*‐related outer surfaces have the limited interfacial contact areas with other monomers owning to the largest curvature as displayed in Figure S3 of Supporting Information (to make the interactions between monomers more liable); consequently, each ferritin building block possesses eight *C_3_* vertices with highly symmetrical geometry and sufficient freedom to arrange.

**Figure 2:**
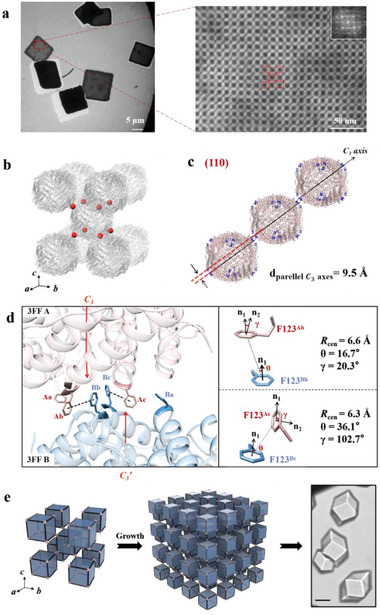
Self‐assembly behavior of 3FF and structural basis of 3D crystalline protein nanocage arrays. a) Self‐assembly of 3FF nanocages into cubic‐like crystalline arrays in solution revealed by TEM. b) The bct packing of the 3D 3FF arrays as revealed by X‐ray crystallography. c) The parallel offset *C_3_* axes (black lines) viewed along (110). F123 residues at the *C_3_* vertex are highlighted in blue. d) The close‐up view of the contact regions near the interacted *C_3_* vertex show that two out of three designed Phe‐Phe pairs are associated with intermolecular π−π interactions. Atomic details of two types Phe–Phe aromatic interactions are shown in the right panel. e) The schematic diagram of 3FF packing pattern in the 3D nanocage arrays. 3FF building blocks are represented by cubes. *C_3_* joints are highlighted as pink spheres. The scale bar of optical micrographs is 50 µm.

We envisioned that 3D crystalline protein nanocage arrays with a body‐centered cubic or tetragonal structure would be constructed if appropriate π−π stacking interactions were introduced along the four *C_3_* rotation axes. Analyses of the crystal structure of HuHF (PDB: 2FHA) revealed that D123 nearby the *C_3_* interfaces is an ideal position for anchoring aromatic motifs, because the side chain of D123 protrudes on the outside surface of the protein cage (Figure S4, Supporting Information), thereby providing sufficient response area for aromatic residues to trigger protein nanocage assembly reaction. To test this idea, we made four ferritin mutants where D123 in native HuHF was replaced by Trp, Tyr, Phe, and His, respectively, through single mutation, which were referred to as 3FW, 3FY, 3FF, and 3FH. Subsequently, they were purified to homogeneity by using nearly the same method as wild‐type ferritin (Figures S5 and S6, Supporting Information), followed by investigation of their assembly behavior in solution by transmission electron microscopy (TEM) with respect to protein concentration, pH and salt concentration. This is because these parameters are expected to govern intermolecular protein interactions, and results were displayed in Table S1 (Supporting Information). Both 3FW and 3FY molecules at 2.0 µm formed heterogeneous aggregates in solutions (20 mm Tris‐HCl, pH 7.5) upon treatment with NaCl (Figure S7, Supporting Information), while 3FF ferritin monomers start to interact with each other, self‐assemble into protein superlattices after 5 min under the same experimental conditions (**Figure** **2**a). 3FF molecules present as the cubic‐like shape from a point view of the *C_4_* axis and are packed with neighboring 3FF monomers at the *C_3_* corners, showing a molecular patterning of body‐centered lattices (Figure 2a). We attributed the large difference in assembly behavior between these three mutants to their different interaction modes, which stems from the aromaticity of Phe distinct from its analogs Trp and Tyr. Agreeing with this view, no protein arrays was obtained with 3FH under identical conditions, and instead protein nanocage molecules are constantly in a monodispersed state because of relatively weak His–His interactions. Similar to 3FH, no protein assembly was likewise observed with wild‐type HuHF (Figure S8, Supporting Information), indicating that the above 3FF assembly is most likely derived from the designed π−π stacking interactions. Taken together, all these findings demonstrate that the formation of 3D crystalline protein arrays exhibit high selectivity for Phe–Phe induced intermolecular interactions.

Interestingly, after 1 h incubation, 3FF ferritin monomers (2.0 µm) self‐assemble into cubic‐like crystalline arrays having different numbers of faces with dozens of micrometers in size in the buffer (20 mm Tris‐HCl, pH 7.5, 200 mm of NaCl) (Figure 1). We named these as “arrays” in order to emphasize that they are constructed based on bottom‐up self‐assembly. In this study, we are attempting to guide the assembly and extension of protein molecules along certain directions by designed intermolecular interactions, which is different with protein crystals that grow under the influence of precipitant and other environmental factors. It also has to be mentioned that 3FF concentrations used here are tenfold lower than those (≈20.0 µm) needed during conventional wild‐type HuHF crystallization procedures that require precipitants such as metal ions or PEGs. Although most of the 3FF crystals displayed low‐resolution diffractions due to their fast growth, we eventually found a suitable crystal that solved the crystal structure at a resolution of 2.90 Å (I422 space group, *a* = *b* = 14.2 nm, *c* = 16.7 nm, *α* = *β* = *γ* = 90°; PDB ID: 8J9M) for structure determination after an exhaustive screen. The X‐ray crystal structure shows that 3FF molecules assemble into body‐centered tetragonal (bct) lattice (Figure 2b), being consistent with our design. Further analyses revealed that the *C_3_* axes of adjacent ferritin molecules are parallel offset and the distance between two nearby *C_3_* axes is 9.5 Å (Figure 2c; Figure S9, Supporting Information). Among three designed Phe‐Phe pairs, two of them at each *C_3_* vertex are involved in the aforementioned π−π interactions between two adjacent ferritin molecules (Figure 2d, we defined an interacting aromatic pair as one for which the distance between phenyl ring centroids (Rcen) is less than 7 Å and/or the shortest inter‐residue carbon‐carbon distance is less than 4.8 Å according to previous reports^[^ ^27^ ^]^), and these two types of π−π interactions are shown in Figure 2d, right panel. The conformation of each π−π interaction is distinguished by the center‐normal angle (θ) and the normal‐normal angle (γ).^[^ ^27d^ ^]^ The F123^Ab^–F123^Bb^ pair takes an offset stacked conformation, while the second pair F123^Ac^–F123^Bc^ shows a T‐shaped conformation. Differently, the third Phe–Phe pair (F123^Aa^–F123^Ba^) are largely mismatched (Figure 2d), causing *C_3_* axes parallel misaligned with equidistance, resulting in the generation of bct lattices rather than body‐centered cubic (bcc) analogs. It is evident that each cubic‐like protein cage interacts with other eight monomers in an isotropic manner along the *C_3_* rotation axes; such isotropic interactions between any adjacent protein molecules extend in 3D space, generating cubic‐like crystals (Figure 2e). Taken together, all these findings demonstrate that 3D crystalline protein nanocage arrays with a bct structure originate from the designed intermolecular π−π interaction and exhibit high selectivity for Phe–Phe pairs rather than its analogs.

### 2.2 Fabrication and Characterization of 1D Crystalline Protein Nanocage Arrays

The formation of the above 3D crystalline protein nanocage arrays requires each 3FF molecule to interact with eight other analogs in an isotropic manner through Phe‐mediated π−π interactions. On one hand, it has been established that π−π interactions are highly sensitive to external stimuli, such as pH.^[^ ^23^ ^]^ This valuable feature raises the possibility that the *C_3_* vertexes modified by three Phe motifs in 3FF represent a kind of fault‐tolerant sites for intermolecular π−π interactions, which meanwhile as a surface‐exposed tripodal joint offer certain freedom to adjust the geometry of flexible π−π interactions, thereby changing the orientation of 3FF assemblies in response to changes in pH values as above excepted. On the other hand, external stimuli such as pH likewise affects the surface charge characteristics of 3FF nanocages, further causing a possible change in their assembly behavior in solution. Indeed, zeta potential measurements and analyses of electrostatic potentials of molecular surfaces calculated by the program APBS (Adaptive Poisson‐Boltzmann Solver) revealed that at pH 9.5, the net charge of ferritin surface becomes more negative as compared with that at pH 7.5, producing larger electrostatic repulsion of entire 3FF building blocks (Figure S10, Supporting Information). In contrast, such pH value decreases the electrostatic potential of the positively charged *C_3_* surface, enabling it less repulsive. Based on these rational considerations and analyses, we envisioned that if the above pH for fabricating the 3D crystalline protein arrays were increased up to 9.5 while keeping ionic strength fixed, it would cause the anisotropic π−π interactions between adjacent 3FF molecules in response to such largely different external stimuli, leading to different crystalline protein arrays from the above observed 3D analogs.

**Figure 3:**
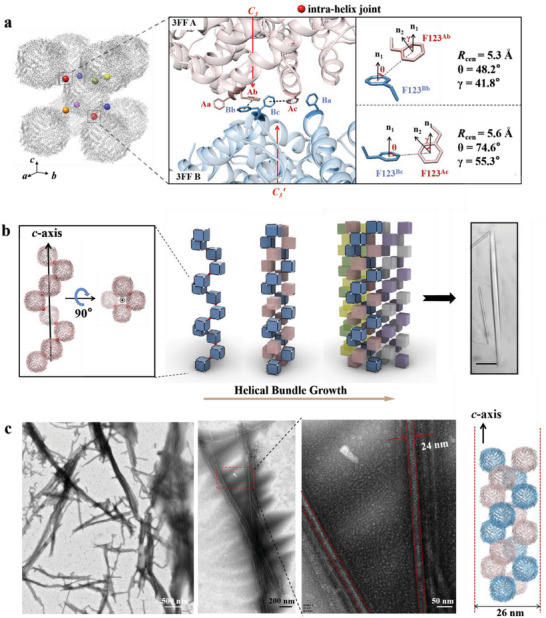
Structural basis of Phe‐Phe mediated 3FF assembly at pH 9.5 plus 200 mm NaCl. a) Left: crystallographically determined molecular arrangement of 3FF nanocages along the designed *C_3_* interfaces. Each 3FF molecule interacts with eight other analogs through six types of Phe‐Phe interactions highlighted in six different colors (joint A‐E indicated by red, yellow, orange, blue, green and purple spheres, respectively). Right: the close‐up view of the contact regions near the interacted *C_3_* vertex of intra‐helix interactions in the 1D rod‐like crystals. Atomic details of each pair of intra‐helix Phe‐Phe interactions are shown enlarged. b) A‐type interactions along the c‐axis as the intra‐helix junctions make the overall structure display a right‐handed helix morphology with four monomers per helical turn. Intertwining of several helices with each other through B to F‐type inter‐helix Phe‐Phe interactions results in the formation of 1D crystalline protein arrays. Intra‐helix joints are highlighted as small red spheres. The scale bar of optical micrographs is 50 µm. c) TEM images of 1D filaments obtained by dissolving rod‐like 3FF crystals at pH 4.0, which are composed of a double helix.

To confirm this view, a self‐assembly behavior of 3FF (2.0 µm) was investigated under the same condition as above except that the pH value of the solution increased to 9.5 (20 mm CAPS). As expected, 3FF molecules assemble into 1D rod‐like crystalline protein arrays in solution (Figure 1), which was not observed in the solution of wide‐type HuHF and other mutants under the same experimental conditions. To explore the relationship between such distinct assembles and designed interactions at the atomic level, the crystal structure of the crystalline protein array was solved at 2.56 Å resolution (I4122 space group, *a* = *b* = 30.1 nm, *c* = 31.6 nm, *α* = *β* = *γ* = 90°; PDB ID: 8JAI). It was observed that each 3FF molecule likewise interacts with eight other 3FF molecules through Phe‐mediated π−π interactions, agreeing with our design. However, unlike the above 3D crystalline protein arrays, the formed π−π interactions consist of six types of junctions (named as interaction/joint A to F respectively) located at the eight *C_3_* vertices (**Figure** **3**a), resulting in a highly asymmetric distribution of Phe‐mediated π−π interactions on each 3FF molecule. Thus, anisotropic π−π interactions have an outstanding performance in the assembly of 3FF molecules upon changing solution pH from 7.5 to 9.5. The arrangement of the 1D crystalline protein arrays mediated by *C_3_* vertex interactions roughly resembles the bct lattice, but the unequal offset distances of parallel *C_3_* axis (Figure S11, Supporting Information) make the structure markedly different from the above 3D crystalline protein arrays where all contacts at vertices are identical.

To quantify the strength of different interactions at *C_3_* surfaces, we determined the interaction energy E_int_ consisting of Coulomb and Lennard–Jones (LJ) contributions performed with GROMACS software, and compared separation distance and interfacial contact areas between two adjacent 3FF building blocks at the same time. The results (Figure S12, Supporting Information) revealed that the A‐type interactions are strongest among six types of Phe‐Phe aromatic interaction, followed by C‐, F‐, D‐, E‐, B‐type analogs (Figure S13, Supporting Information). Joint‐A is mediated by two pairs of Phe‐Phe interactions, one (F123^Ab^‐F123^Bb^) of which possesses a T‐shape conformation, and the other (F123^Ac^–F123^Bc^) is offset stacked (Figure 3a). Structure analyses revealed that the 3FF monomers jointed by the A‐type interactions penetrate along the *c*‐axis in a right‐handed‐helix manner with four monomers per helical turn (Figure 3b). A bundle of such protein nanocage helixes join together along the *ab*‐plane through relatively weak π−π interactions, resulting in the production of the 1D rod‐like crystals. Consistent with the above 1D assembly analyses, TEM images of intermediate assemblies captured when dissolving the rod‐like crystals at pH 4.0 (100 mm sodium acetate) show a fine filament shape with a width of ≈24 nm (Figure 3c), which is close to a double helix, further confirming that the structure of the 1D crystalline arrays possesses the long‐range orientational ordering governed by the stronger π−π interactions along its elongate direction. Collectively, all these results demonstrate that with Phe‐decorated 3FF molecules as building blocks, pH is able to serve as a switch to control conversion of isotropic intermolecular π−π interactions to anisotropic interactions, finally realizing shape tunability to its 1D analogs.

### 2.3 Fabrication and Characterization of 2D Crystalline Protein Nanocage Arrays

**Figure 4:**
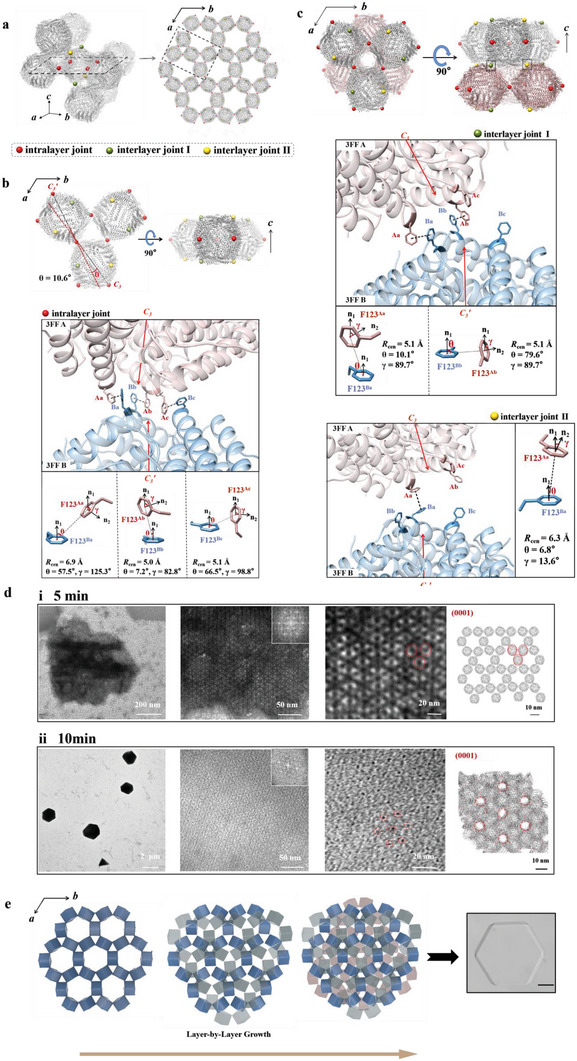
Structural basis of Phe‐Phe mediated 3FF assembly at pH 9.5 plus 800 mm NaCl. a) Crystallographically determined packing pattern of 3FF nanocages along the designed *C_3_* interfaces. Left: three different intermolecular interactions are distributed on the eight vertices of each cubic‐like 3FF monomer in the crystals. Intralayer joints are highlighted as red spheres, and interlayer joints are highlighted as green and yellow ones. Right: the arrangement of 3FF monomers in the *ab*‐plane of the equilateral‐hexagon‐like crystals. b) Structural basis of intralayer interactions and the close‐up view of the contact regions near the interacted *C_3_* vertex and atomic details of each pair of Phe–Phe interaction of intralayer interactions. c) Structural basis of interlayer interactions along the *c*‐axis, and interlayer joints I and II are highlighted as green and yellow spheres respectively. The close‐up view of the contact regions near the interacted *C_3_* vertex and atomic details of each pair of Phe‐Phe interaction of interlayer interactions are shown. d) TEM views of the kinetics of 2D crystalline 3FF arrays. TEM images of growing protein nanocage arrays at (row i) 5 min and (row ii) 10 min. Insert: Fast Fourier transform image. Right: structural model of the 2D crystalline protein arrays on the basis of TEM images. e) Crystallographically determined molecular arrangement in 2D 3FF sheets, viewed normal to the *ab*‐plane of crystalline 3FF arrays. The hexagonal‐layered packing arrangement of 3FF molecules generates equilateral‐hexagon‐like crystals. The scale bar of optical micrographs is 50 µm.

Bear in mind that besides pH, π−π interactions are also sensitive to ionic strength.^[^ ^24^ ^]^ Therefore, to elucidate effects of ionic strength on the assembly behavior of 3FF molecules in solution, we fixed pH at 9.5 and carried out a broader screen of salt concentration (>200 mm NaCl). Excitingly, 3FF molecules self‐assembled into 2D equilateral‐hexagon‐like crystalline protein arrays in a solution (2.0 µm of 3FF, 20 mm CAPS, pH 9.5, 800 mm NaCl) upon incubation for 1 h, whereas wide‐type HuHF and other mutants could not form such arrays with identical experimental conditions, demonstrating that our designed π−π interactions play a key role in the above self‐assembly process. To elucidate the detailed structure of the above 2D crystalline protein nanocage arrays at the atomic level, we determined their crystal structure at 2.72 Å resolution featuring a hexagonal packing (H32 space group, *a* = *b* = 26.1 nm, *c* = 32.0 nm, *α* = *β* = 90°, *γ* = 120°; PDB ID: 8J9L). Consistent with our design, each 3FF molecule keeps interaction with eight other molecules (**Figure** **4**a) along the *C_3_* axes through the Phe‐mediated π−π interactions in the crystals. However, different from the aforementioned 3D and 1D assembles, the intermolecular π−π interactions responsible for the formation of the crystals are composed of two kinds of interactions (intralayer and interlayer interactions), demonstrating another kind of anisotropic π−π interactions for 3FF assembly in response to different external stimuli (pH 9.5, 800 mm of NaCl).

Figure 4b displays the detail of the intralayer joint that consists of three heterogeneous T‐shape π−π interactions, resulting in the intersected *C_3_* axes with an intersection angle of 10.6°. Each layer is attached to the neighboring layer at other four *C_3_* symmetrical vertices in two weaker forms, interlayer joints I and II as shown in Figure 4c. Joint I corresponds to two identical T‐shape π−π interactions, while joint II is composed of a pair of offset stacked π−π interaction. Subsequently, with the *C_3_* symmetrical vertices as the only interframe connectivity, we also determined interaction energy of two *C_3_* pore interfaces of interacted ferritin monomers, and compared their separation distance and interfacial contact areas (Figure S14, Supporting Information). Calculated results demonstrate that the intralayer interaction owns the highest binding affinity, largest buried surface area and the shortest distance between two *C_3_* pores, suggesting that the intralayer interaction is the main driven force for the equilateral‐hexagon‐like morphology of the 2D crystalline lattices.

To obtain more information about the 2D arrays, their formation kinetics was investigated by TEM which revealed a gradual increase in the 2D size. At an early stage, the thinly layered structure was formed (Figure 4d) after incubation for 5 min. Ferritin monomers resembled the thin ellipses has been viewed along the twofold axis, and translated periodically in hexagonal layers. At 10 min, the layers got thicker (Figure 4d) and thus only the honeycomb‐like porous structure could be distinguished. The pattern of the crystalline protein arrays observed is consistent with the (0001) plane of the crystal structure.

What most interests us about this 2D crystalline lattice is that anisotropic Phe‐Phe interactions enable 3FF molecules to pack preferentially in the *ab*‐plane, finally producing equilateral‐hexagon‐like crystals. As 3FF self‐assembly is mediated by easily identifiable intermolecular interactions in all three dimensions, we can correlate crystal morphology with the strength of these interactions. The strong planar π‐interactions could enhance the growth of monomers along the hexagonal layer in the *ab*‐plane, so the 2D sheets observed under fast nucleation conditions were equilateral‐hexagon. The slipped‐parallel hexagonal layers stack along the *c*‐axis by contacts of weak π−π interactions, so the final crystalline arrays are thinnest along the *c*‐axis (Figure 4e).

### 2.4 Interconversion of 3D, 2D, and 1D Crystalline 3FF Protein Nanocage Arrays

A major goal in synthetic biomimetic materials is to apply chemical design at the atomic/molecular scale to create hierarchical assemblies that respond to external stimuli such as pH by changing their physical or chemical structure in response to environmental cues.^[^ ^5c^ ^]^ Considering that the newly fabricated three crystalline protein assemblies in solution are constructed from the same protein nanocage building blocks by the designed π−π noncovalent interactions, we wonder whether they can be shifted into each other in response to external stimuli. To this end, we optimized the solution condition and found that all three kinds of crystalline lattices can disassemble back into monomers when the solution pH is lowered to 4.0. Such disassembled protein nanocages from one kind of protein nanocage array are available to reassemble into other two different protein arrays after corresponding solution conditions were established, indicating the reversibility and tunability of 3FF crystalline lattices (Figure S15, Supporting Information). Thus, these three distinct protein assemblies are conformationally switchable, and their mutual transformation can be tuned by external chemical stimuli with discrete 3FF nanocages as intermediates.

## 3 Conclusion

In this study, we present an engineering strategy by combining diverse, tunable Phe‐mediated interactions with the symmetrical geometry of protein nanocage building blocks to develop different topological connections inside the lattices. With this strategy, we constructed shape‐anisotropic crystalline protein nanocage arrays by using the same building block which was easily fabricated by single mutation. Notably, shape transformation can be carried out with these different crystalline protein arrays in response to environmental cues such as pH and ionic strength, which could confer immense potential in producing other protein functional materials and recapitulate the ability of protein nanocages to assume distinct conformational states.

This work highlights three important concepts previously unrecognized. First, Phe‐mediated π−π interactions can capture all the salient features of interprotein interactions (specificity, directionality, symmetry and reversibility) on a much small surface, and thus require “minor design”. The unique π−π interactions endows them with the distinct ability to mediate protein assemblies differing from other interactions. Besides, the long‐range π−π interactions occurring within 7 Å give the protein molecules more freedom to move or rotate dynamically as compared with other short‐range interactions, such as hydrogen bonds and disulfide bonds, whose distances usually don't exceed 3 Å.^[^ ^28^ ^]^ Second, the Phe‐involved π−π interactions exhibit much high flexibility in response to external stimuli, which could be used to create dynamic, transmutable protein materials. Such flexibility in intermolecular interactions plays a key role in our fabricated three types of crystalline protein nanocage arrays based on the fact that more than ten different kinds of π−π interaction conformations occur between two adjacent 3FF protein molecules under different solutions conditions. This interesting property makes it completely different from metal‐coordination interaction which is rigid as the stereochemical preferences of metal ions, thereby dictating the symmetry and structures of protein assemblies.^[^ ^29^ ^]^ Third, a combination of Phe‐Phe interactions and multiple, symmetric interaction sites could be explored to construct shape‐ and direction‐anisotropic crystalline protein materials. However, further exploration of using other aromatic amino acid residues to construct assemblies holds significant potential.

In general, our study underscores the utility of the flexibility of π−π interaction and the symmetry of building blocks in creating protein materials that possess both high order and tunability. The idea used here may also be suitable for a number of known shell‐like proteins or proteins with high symmetry because they likewise possess multiple interaction sites symmetrically distributed on the exterior surface nearby the different rotation axes.

## Conflict of Interest

The authors declare no conflict of interest.

## Supporting information

Supporting Information

## Data Availability

The data that support the findings of this study are available in the supplementary material of this article.
