# Supplementary material for: Shape‐Anisotropic Assembly of Protein Nanocages with Identical Building Blocks by Designed Intermolecular π–π Interactions
Source: Adv Sci (Weinh). 2023 Oct 23;10(35):2305398. doi: 10.1002/advs.202305398 (PMC10724428; doi:10.1002/advs.202305398)
Supplement: Supplementary file 1 — Supporting Information [file ADVS-10-2305398-s001.pdf]

ADVANCED SCIENCE
Open Access

Supporting Information

for *Adv. Sci.*, DOI 10.1002/advs.202305398

Shape-Anisotropic Assembly of Protein Nanocages with Identical Building Blocks by Designed Intermolecular π–π Interactions

Xuemin Chen, Tuo Zhang, Hanxiong Liu, Jiachen Zang, Chenyan Lv, Ming Du and Guanghua Zhao**

Supporting Information
©Wiley-VCH 2021
69451 Weinheim, Germany

Shape-anisotropic Assembly of Protein Nanocages with Identical Building Blocks by Designed Intermolecular π–π Interactions

Xuemin Chen, Tuo Zhang, Hanxiong Liu, Jiachen Zang, Chenyan Lv,* Ming Du, and Guanghua Zhao*

Abstract: Protein lattices that shift the structure and shape anisotropy in response to environmental cues are closely coupled to potential functionality. However, to design and construct shape-anisotropic protein arrays from the same building blocks in response to different external stimuli remains challenging. Here, by a combination of the multiple, symmetric interaction sites on the outer surface of protein nanocages and the tunable features of phenylalanine-phenylalanine interactions, we report a protein engineering approach to construct a variety of superstructures with shape anisotropy, including 3D dodecahedral, 2D hexagonal layered and 1D needle-like crystalline protein nanocage arrays by using one single protein building block. Notably, the assembly of these crystalline protein arrays is reversible, which can be tuned by external stimuli (pH and ionic strength). The anisotropic morphologies of the fabricated macroscopic crystals can be correlated with the Å-to-nm scale protein arrangement details by crystallographic elucidation. These results enhance our understanding of the freedom offered by an object’s symmetry and inter-object π−π stacking interactions for protein building blocks to assemble into direction- and shape-anisotropic biomaterials.

Table of Contents

Experimental Procedures
Protein expression and purification .... **Error! Bookmark not defined.**
Preparation of 3FF assemblies .... **Error! Bookmark not defined.**
Polyacrylamide gel electrophoresis. .... **Error! Bookmark not defined.**
Data collection, and structure determination of 3FF crystalline arrays .... **Error! Bookmark not defined.**
Transmission electron microscopy (TEM) imaging .... **Error! Bookmark not defined.**
Zeta potential analyses .... **Error! Bookmark not defined.**
Electrostatic potentials by the Poisson−Boltzmann equation .... **Error! Bookmark not defined.**
Interaction energy calculations .... **Error! Bookmark not defined.**
Results and Discussion
Supplementary Figures
Figure S1 4
Figure S2 5
Figure S3 6
Figure S4 7
Figure S5 8
Figure S6 9
Figure S7 10
Figure S8 11
Figure S9 12
Figure S10 .... 1**Error! Bookmark not defined.**
Figure S11 14
Figure S12 15
Figure S13 16
Figure S14 17
Figure S15 18
Supplementary Table
Table S1 19
Table S1 20
References .... **Error! Bookmark not defined.**
Author Contributions .... **Error! Bookmark not defined.**

Experimental Procedures

Protein Expression and Purification

The gene encoding of ferritin mutants (3FW, 3FY, 3FF, and 3FH) and wild-type HuHF was built in a pET-3a vector, respectively. The expression of wild-type HuHF, 3FW, 3FY, 3FF, and 3FH were induced in Rosetta (DE3) in lysogeny broth by 0.1 mM IPTG (isopropyl-β-D-1-thiogalactopyranoside) at $OD_{600\,nm}$ ~ 0.6, and then the cells were cultured at 37 °C for additional 8 h before harvest. Subsequently, cells were pelleted and resuspended in 20 mM Tris-HCl, pH = 7.5. The cells were lysed for 15 min on ice with ultrasonic, and the lysates were centrifuged for 10 min at 10000 rpm, 4 °C to collect the supernatant. The wild-type HuHF and mutants were precipitated by 60% and 40% $(NH_4)_2SO_4$ respectively, and the resulting precipitates were re-dissolved by dialysis into 20 mM Tris-HCl, pH = 7.5. The target protein was purified followed by a gel filtration column (Superdex 200pg 16/60, GE Healthcare).

Preparation of 3FF Assemblies

After purification, 3FF molecules remain as a monomer in the absence of NaCl. For 3D crystalline arrays, 20 mM Tris–HCl (pH 8.0) was used as buffer and 200 mM NaCl was required to induce 3FF (2.0 μM) self-assembly. 3D crystalline arrays were prepared by adding 200 mM NaCl to 3FF solution (2.0 μM, 20 mM Tris–HCl pH 7.5). After being stirred for several minutes, resulting mixture was incubated at 20 °C. To optimize the assembly conditions, different concentrations of NaCl were tested over a range of 0-800 mM. Similarly, 1D and 2D crystals were prepared by adding 200 mM and 800 mM NaCl to 3FF solution (2.0 μM, 20 mM CAPS pH 9.5). After gently shaking for a few minutes, the resulting mixture was incubated at 20 °C.

Disassembly and Interconversion of 3D, 2D, and 1D 3FF Crystalline Arrays

3FF Assemblies disassemble into monomers when treated with 200 mM acetate buffer (pH 4.0). To convert these protein nanocages into other crystalline arrays, the above protein solution was dialyzed against 20 mM Tris-HCl (pH = 7.5) for three times to move salt and change pH. The protein was concentrated to 2.0 μM, and subjected to re-assembly into a second and third arrays by regulating the solution conditions to match the corresponding conditions screened in the 'Preparation of 3FF Assemblies' section.

Polyacrylamide Gel Electrophoresis

The purity and molecular weight of protein samples were estimated by PAGE. Gel electrophoresis under denaturing conditions was carried out using a 15% polyacrylamide–SDS gel as reported by Laemmli, and samples need to be heated in a water bath for 5 min. For native PAGE, a 4–20% polyacrylamide gradient gel was used and run at 5 mA for 10 h at 4 °C. Gels were stained with Coomassie brilliant blue R250.

Data Collection, and Structure Setermination of 3FF Crystalline Arrays

X-ray diffraction data were collected at BL18U1 or BL02U1 at Shanghai Synchrotron Radiation Facility (SSRF) section. The diffraction datasets were indexed, integrated, and scaled by HKL3000 or XDS. The structures were solved through molecular replacement by Phaser-MR in Phenix using a mutant MF structure (PDB ID: 2FHA) as the search template. Iterative model building and refinement were conducted in COOT [1] and Phenix, [2] and all the crystallographic statistics were shown in Supplementary Table 2. All the figures of protein structures were generated by Chimera.[3]

Transmission Electron Microscopy (TEM) Imaging

A drop of the protein sample (ferritin monomers or assemblies) was deposited on a carbon-coated copper grid for 4 min incubation. Then the excess solution was removed with filter paper and stained using 2% uranyl acetate for 4 min. Transmission electron micrographs were imaged at 80 kV with a Hitachi H-7650 transmission electron microscope.

Zeta Potential Analyses

Zeta potential analysis were conducted with a Zetasizer Nano. Samples containing 0.1 mg/mL protein were measured in 800 μL of buffer at 25 °C. The sample pH was adjusted by adding 50 mM Tris–HCl buffer (pH 7.5), 50 mM CAPS buffer (pH 9.5).

Electrostatic Potentials by the Poisson−Boltzmann Equation

The electrostatic potentials for 3FF (PDB ID: 8J9M) were calculated by the program APBS (Adaptive Poisson−Boltzmann Solver). Briefly, the protonation states of all amino acid residues with ionizable side chains were assigned at different pH with PROPKA 3.0 implemented in the PDB2PQR web server (http://server.poissonboltzmann.org/pdb2pqr). It generated the output as a PQR file (containing atomic radii and charges instead of B-factor entries and occupancy, respectively) with the PDB file as the input, using the PARSE force field. The ferritin protein was placed in the cubic space grid with a minimum grid size of 0.5 Å. The electrostatic potential values, solved using the linear Poisson−Boltzmann equation, are expressed in the units of k_BT/e, where k_B is the Boltzmann constant, T is the temperature, and e is the electronic charge.

Interaction Energy Calculations

The molecular dynamics (MD) simulations were performed to determine the interaction energies between 3FF monomers *via* different C_3 vertical interactions in 1D (PDB ID: 8JAI) and 2D (PDB ID: 8J9L) arrays. All simulations were performed with GROMACS software package, version 2023.1. [4] The scheme developed for the Martini model was used. [5] The 3FF monomer complexes were centered in a cubic box of size 216 × 217 × 221 Å^3, solvated with water, and Na^+ and Cl^- added at a concentration of 200 mM for 1D structure and

800 mM for 2D structure while at the same time neutralizing the system. The energy of the systems was minimized *via* the steepest descent algorithm. Afterwards, the systems were equilibrated, first in the NVT ensemble (i.e., with a constant number of molecules, volume, and temperature) for 3 ns and second for 1 ns in the NpT ensemble at 293 K and 1.0 bar. Their interaction energy [6] $\boldsymbol{E}_{\mathbf{int}} = \boldsymbol{E}_{\mathbf{Coul}} + \boldsymbol{E}_{\mathbf{LJ}}$ consisting of Coulomb and Lennard-Jones (LJ) contributions was determined, which was accomplished by rerunning the simulation using gmx mdrun -rerun to obtain the energies, which were processed using gmx energy to calculate E_{Coul} and E_{LJ} between the 3FF monomers.

Statistical Analysis
All data were analyzed and plotted using Origin 2019. The data of zeta potential of 3FF were presented as means ± standard deviation and analyzed with a sample size of n = 3. The results in Figure S10 were analyzed using a one-way ANOVA with Tukey’s post-hoc test. The significance values are indicated with **** as $p < 0.0001$.

Results and Discussion

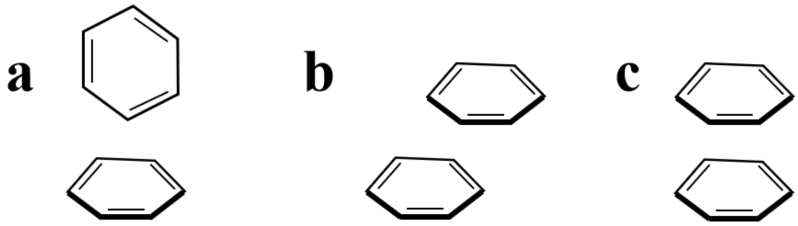

Figure S1. Three typical aromatic stacking arrangements. (a) Edge-to-face. (b) Parallel offset. (c) Parallel face-centred.

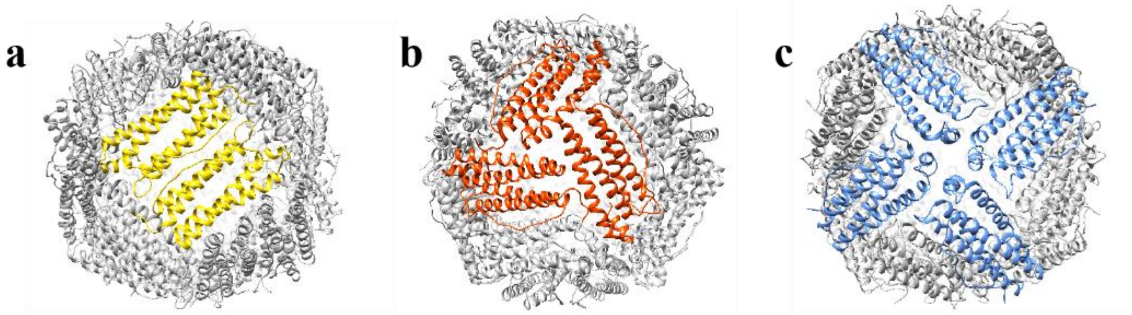

Figure S2. Schematic representation of three kinds of intersubunit interfaces in ferritin. (a) C_2 interfaces. (b) C_3 interfaces. (c) C_4 interfaces.

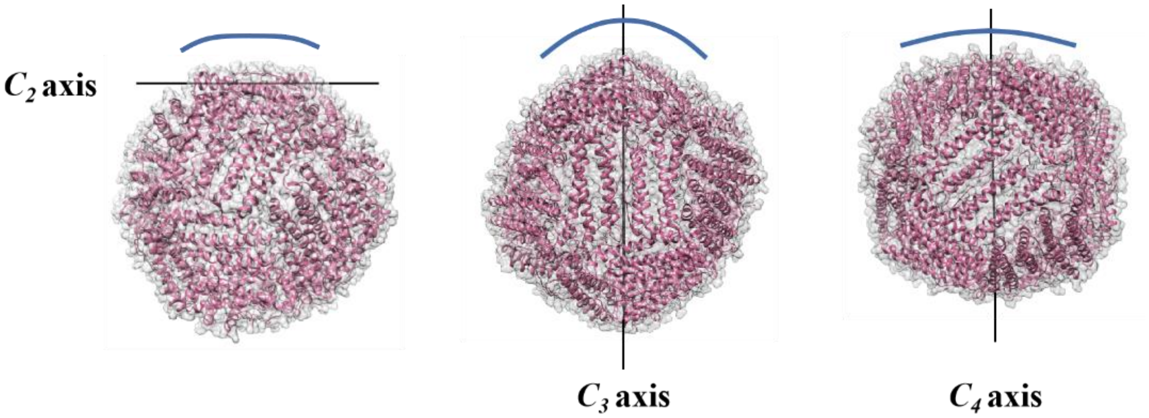

Figure S3. The outer surfaces related to the C_2, C_3, and C_4 axes show different curvatures. The outer surface around the C_3 has the largest curvature.

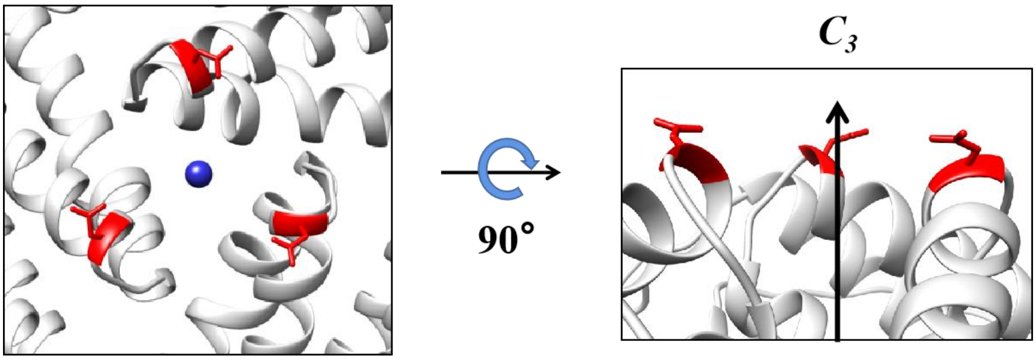

Figure S4. Location and orientation of D123 in wild-type HuHF. D123 residues are nearby the C_3 rotation axes, which are highlighted in red. The center of the C_3 pore is represented by blue sphere.

```
wild type HuHF  MTTASTSQVRQNYHQDSEAAINRQINLELYASYVYLSMSYYFDRDDVALKNFAKYFLHQSHEE
3FF             ---------------------------------------------------------------
3FW             ---------------------------------------------------------------
3FY             ---------------------------------------------------------------
3FH             ---------------------------------------------------------------

wild type HuHF  REHAEKLMKLQNQRGGRIFLQDIKKPDCDDWESGLNAMECALHLEKNVNQSLLELHKLATDK
3FF             ------------------------------------------------------------F-
3FW             ------------------------------------------------------------W-
3FY             ------------------------------------------------------------Y-
3FH             ------------------------------------------------------------H-

wild type HuHF  NDPHLCDFIETHYLNEQVKAIKELGDHVTNLRKMGAPESGLAEYLFDKHTLGDSDNES
3FF             ----------------------------------------------------------
3FW             ----------------------------------------------------------
3FY             ----------------------------------------------------------
3FH             ----------------------------------------------------------
```

Figure S5. Sequence alignment of wild-type HuHF, 3FF, 3FW, 3FY, and 3FH.

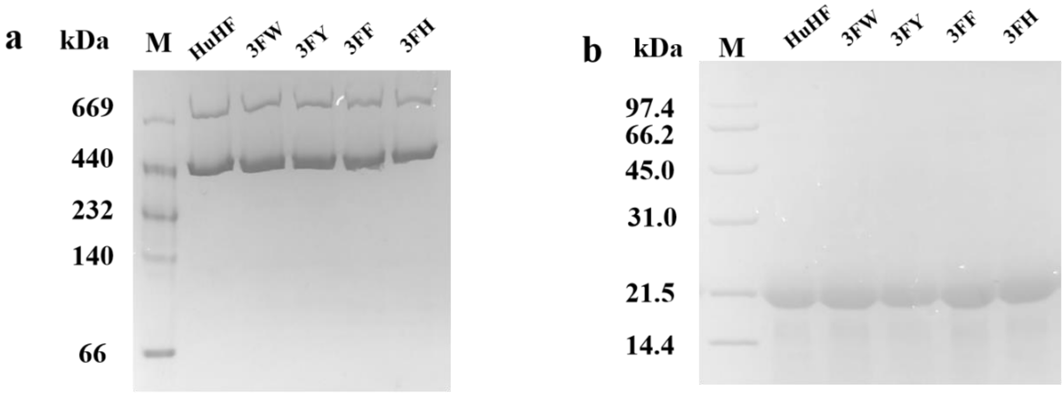

Figure S6. Characterization of wild type and four mutants (3FW, 3FY, 3FF, and 3FH) by native- and SDS-PAGE. (a) Native-PAGE and (b) SDS-PAGE analyses. Lane M in (a) and (b), protein markers and their corresponding molecular masses.

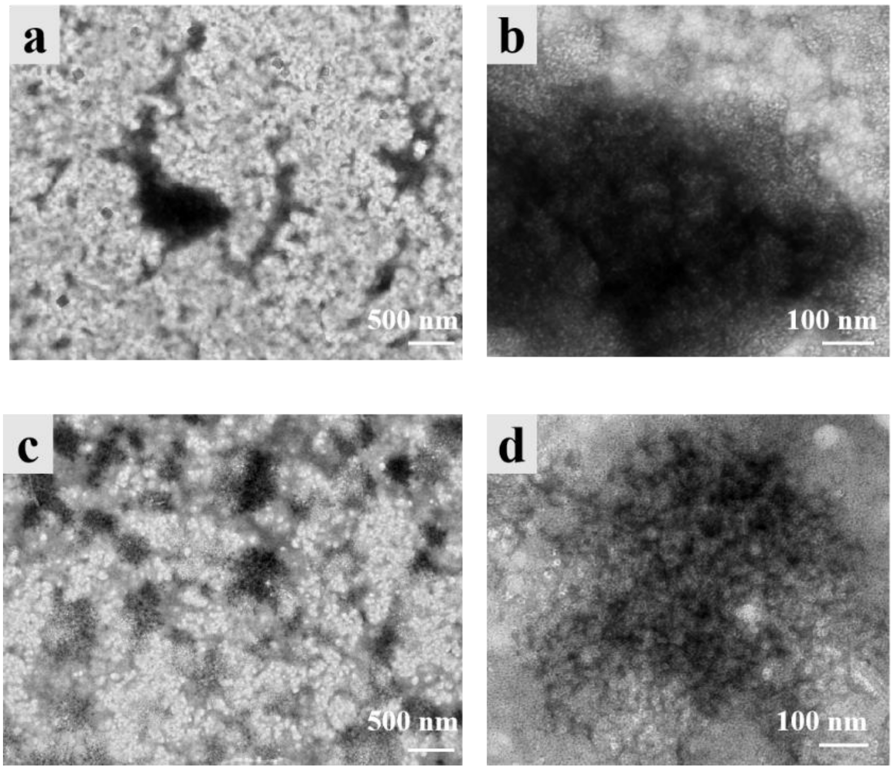

Figure S7. TEM images of (a, b) 3FW and (c, d) 3FY aggregates in 20 mM Tris-HCl, pH 7.5 containing 200 mM NaCl.

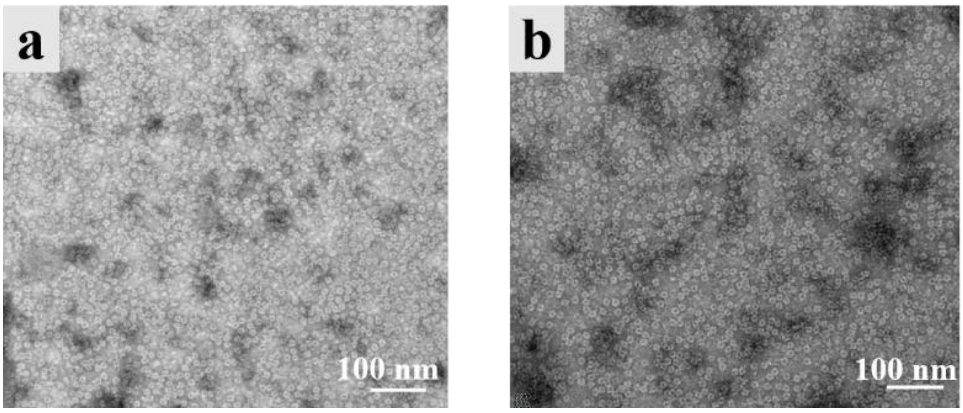

Figure S8. TEM images of (a) wild-type HuHF and (b) 3FH in 20 mM Tris-HCl, pH 7.5 containing 200 mM NaCl.

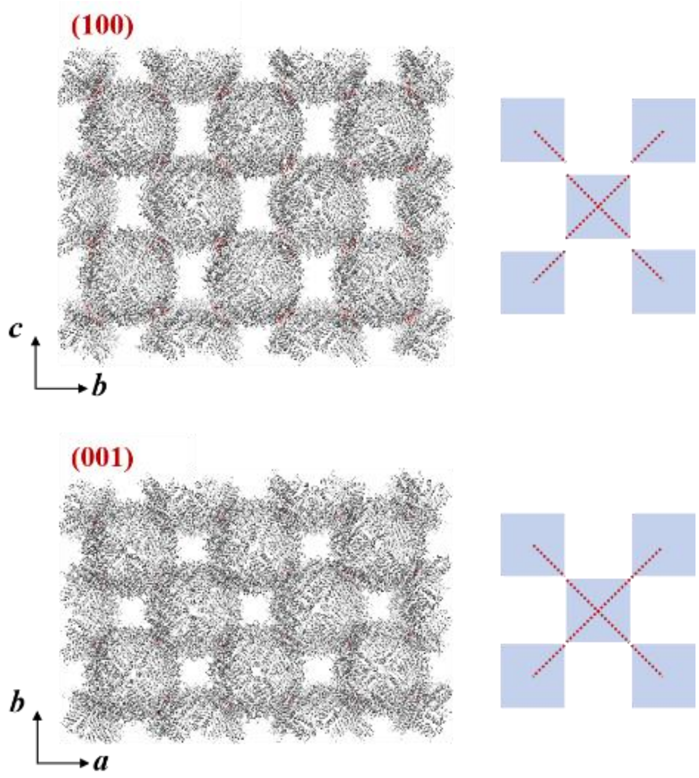

Figure S9. sc-XRD structure of 3D lattice viewed along (a) (100) and (b) (001). Dashed lines are projections of C_3 axes.

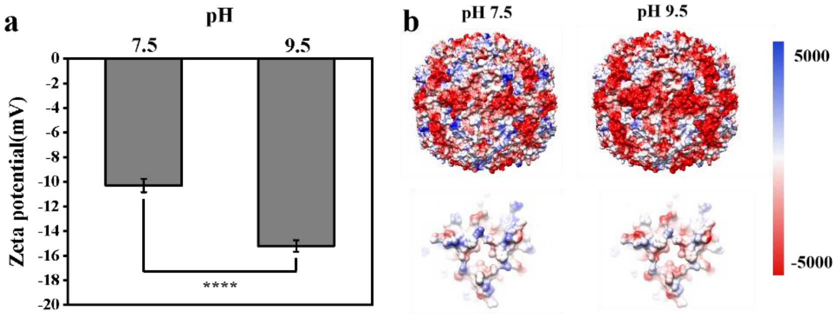

Figure S10. (a) Zeta potential of 3FF at different pH (means ± standard deviation, n = 3, ****$p < 0.0001$). Results were analyzed using a one-way ANOVA with Tukey's post-hoc test. (b) Effect of pH on the surface electrostatics of 3FF. Poisson−Boltzmann electrostatic potential mapped onto molecular surfaces of a monomer and the C_3 surfaces. The surface electrostatics (electrostatic potential expressed in the units of ± 5 $k_B T/e$) is generated using the APBS tool, where red, white, and blue patches indicate the presence of negatively, neutrally, and positively charged amino acid residues, respectively.

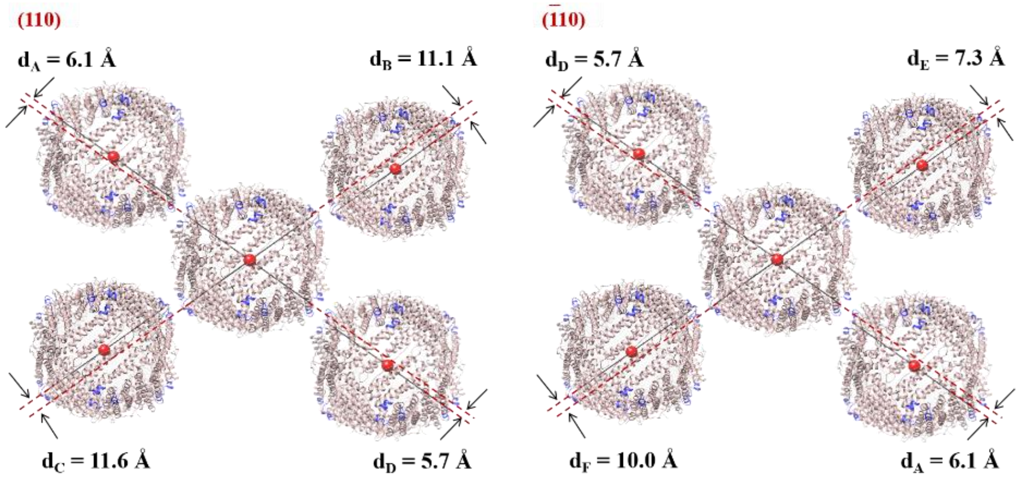

Figure S11. The parallel offset C_3 axes (black lines) viewed along (110) and ($\bar{1}$10). F123 are highlighted in blue.

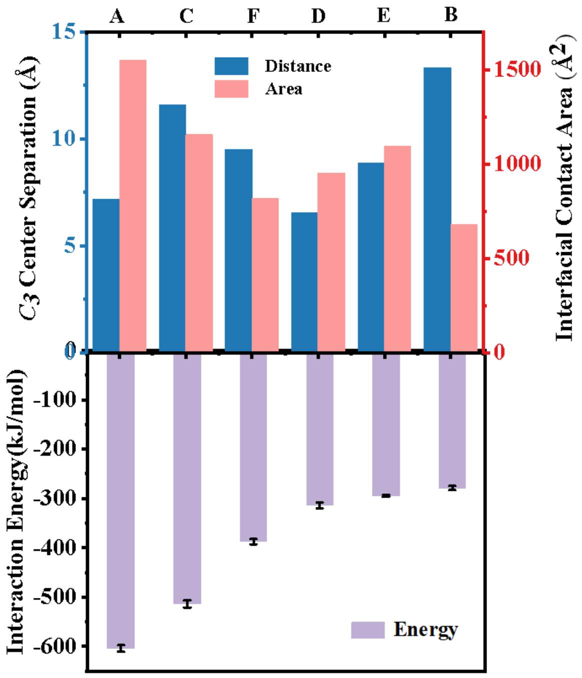

Figure S12. Comparison of interaction energy (E_{int}) consisting of Coulomb and Lennard-Jones (LJ) contributions, separation distances and interfacial contact areas of interacted C_3 interfaces (joint A to F) in 1D lattice.

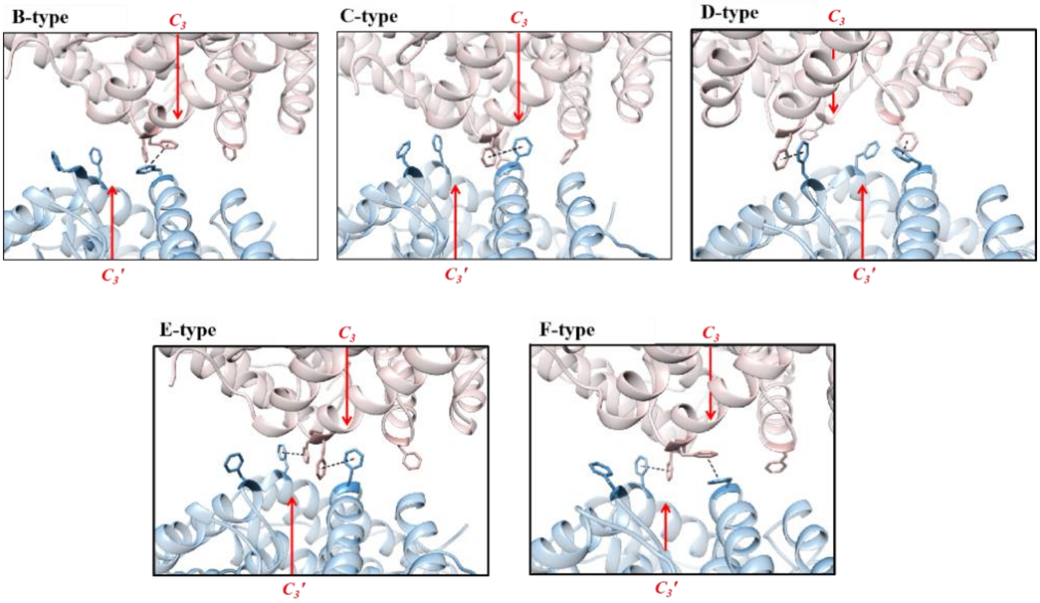

Figure S13. Closeup views of the intermolecular interactions at joint B-F in 1D lattice.

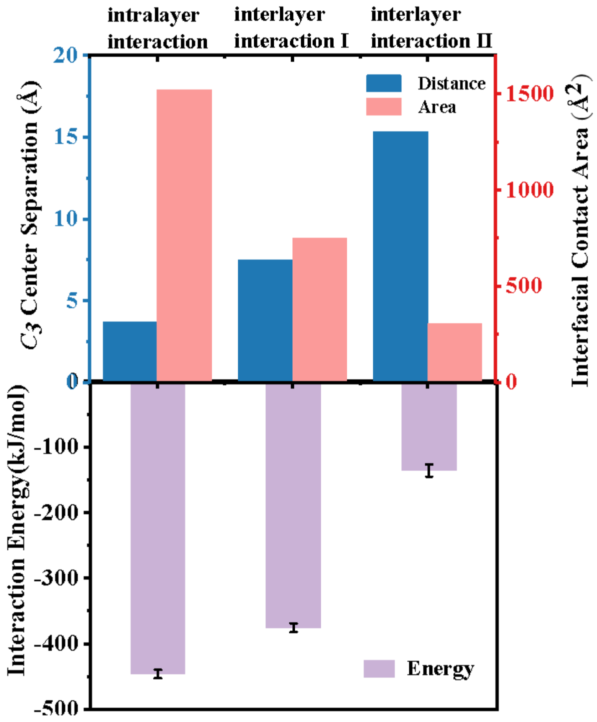

Figure S14. Comparison of interaction energy (E_{int}) consisting of Coulomb and Lennard-Jones (LJ) contributions, separation distances and interfacial contact areas of interacted C_3 interfaces 2D lattice.

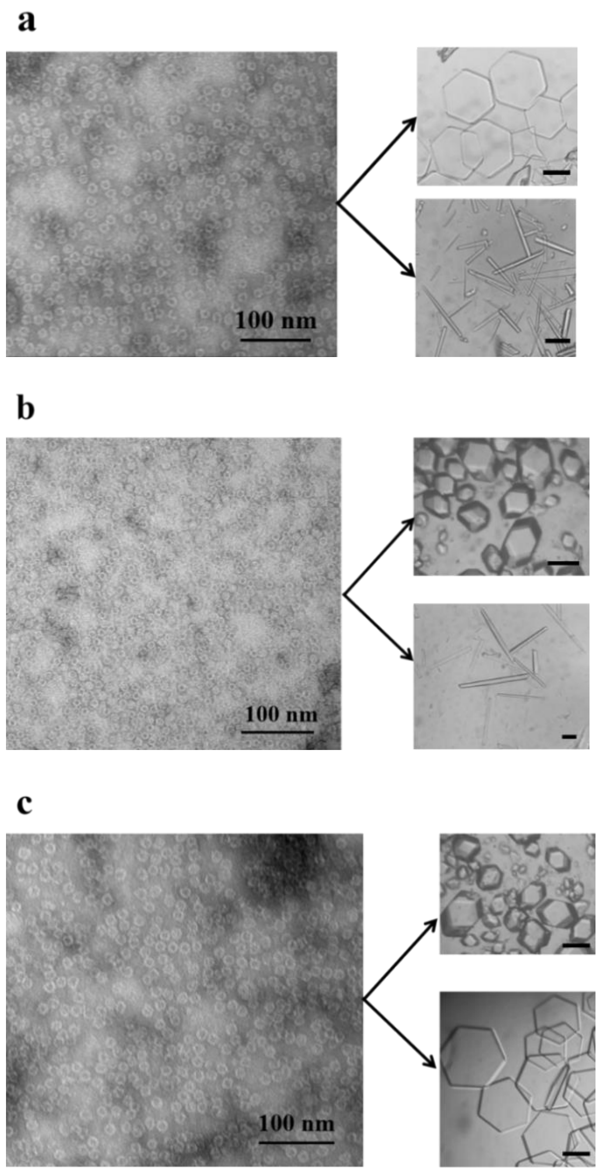

Figure S15. Disassembled 3FF (a) 3D, (b) 2D and (c) 1D crystalline lattices rearranged into other lattices. The scale bar of optical micrographs is 100 μm.

Table S1. Different assembly behaviors of wide-type HuHF, 3FW, 3FY, 3FF and 3FH at different pH values and salt concentrations

Wide-type HuHF and 3FH	pH 7.50	pH 8.50	pH 9.50
0-800 mM NaCl	M	M	M

3FW and 3FY	pH 7.50	pH 8.50	pH 9.50
0 mM NaCl	M	M	M
≥200 mM NaCl	P	P	P

3FF	pH 7.50	pH 8.50	pH 9.50
0 mM NaCl	M	M	M
200 mM NaCl	3D	3D	1D
800 mM NaCl	P	P	2D

P, precipitates; M, monomers; 1D, 1D assembly; 2D, 2D assembly; 3D, 3D assembly.

Table S2. Crystallographic statistics.

**Crystal**	3D assembly (8J9M)	1D assembly (8JAI)	2D assembly (8J9L)
**Data collection**			
Wavelength (Å)	0.97892	0.97892	0.97892
Space group	I422	$I4_122$	H32
Unit cell			
a, b, c (Å)	143.331, 143.331, 166.663	301.66 301.66 316.99	261.696 261.696 320.511
α, β, γ (°)	90.0, 90.0, 90.0	90.0, 90.0, 90.0	90.0, 90.0, 120.0
Resolution [a] (Å)	28.25-2.90	28.18-2.56	29.26-2.50
Multiplicity [a]	1.1 (1.1)	2.0 (2.0)	2.0 (2.0)
Completeness [a] (%)	98.25 (88.21)	99.89 (99.98)	97.24 (79.02)
$I/\sigma I$ [a]	45.70 (36.28)	7.56 (1.22)	4.00 (0.64)
$CC_{1/2}$ [b]	1 (1)	0.996 (0.43)	0.412 (0.702)
**Refinement**			
Reflections used in refinement	19248 (1684)	231076 (22910)	140647 (11346)
Reflections used for R-free	1026 (97)	11622 (1205)	1961 (158)
Macromolecules	4248	33981	16992
Ligands	8	18	16
Solvent	17	2	65
R_{work} [c]	0.2027	0.3344	0.2473
R_{free}	0.2416	0.3686	0.2892
Wilson B-factor (Å^2)	48.65	47.19	14.02
R.m.s. deviations			
Bond lengths (Å)	0.009	0.012	0.009
Bond angles (°)	1.17	1.30	1.10
Ramachandran plot (%)			
Favored	96.47	85.12	97.35
Allowed	3.14	12.60	2.55
Outliers	0.00	2.28	0.10

[a] Highest resolution shell is shown in parentheses.
[b] CC1/2 is the correlation coefficient of the half datasets.
[c] $R_{work} = \sum_{hkl} \mid |F_{obs}| - |F_{calc}| \mid / \sum_{hkl} |F_{obs}|$, where F_{obs} and F_{calc} is the observed and the calculated structure factor, respectively. R_{free} is the cross-validation R factor for the test set of reflections (5% of the total) omitted in model refinement.

References

[1] P. Emsley, B. Lohkamp, W. G. Scott, K. Cowtan, *Acta Crystallogr. D Biol. Crystallogr.* **2010**, *66*, 486-501.
[2] R. Torice, A. J. Muñoz-Pajares, *Appl. Plant Sci.* **2015**, *5*, 1400104.
[3] E. F. Pettersen, T. D. Goddard, C. Huang, G. S. Couch, *J. Comput. Chem.* **2004**, *25*,1605-1612.
[4] B. Hess, C. Kutzner, D. van der Spoel, E. Lindahl, *J. Chem. Theory Comput.* **2008**, *4*, 435-447.
[5] a) S. J. Marrink, A. H. de Vries, A. E. Mark, *J. Phys. Chem. B* **2004**, *108*, 750-760; b) S. J. Marrink, H. J. Risselada, S. Yefimov, D. P. Tieleman, A. H. de Vries, *J. Phys. Chem. B* **2007**, *111*, 7812-7824.
[6] J. Loschwitz, A. Jäckering, M. Keutmann, M. Olagunju, R. J. Eberle, M. A. Coronado, O. O. Olubiyi, B. Strodel, *Bioorg. Chem.* **2021**, *111*, 104862.

Author Contributions

G.Z. and C.L. conceived and directed the project and wrote the paper. X.C. designed and performed experiments, analyzed data, and co-wrote the paper. H.L. M.D. performed molecular dynamics simulations. T.Z., J.Z. performed the experiments and co-wrote the paper.
